# Mediastinal Germ Cell Tumor-associated Histiocytic Proliferations Treated With Thalidomide Plus Chemotherapy Followed by Alemtuzumab-containing Reduced Intensity Allogeneic Peripheral Blood Stem Cell Transplantation: A Case Report

**DOI:** 10.1097/MD.0000000000002515

**Published:** 2016-01-15

**Authors:** Li-Hua Fang, Li-Sun Shih, Pei-Ing Lee, Wei-Ting Chen, Rong-Long Chen

**Affiliations:** From the Department of Pharmacy, Koo Foundation Sun Yat-Sen Cancer Center, Taipei, Taiwan (L-HF); Department of Pathology, Koo Foundation Sun Yat-Sen Cancer Center, Taipei, Taiwan (L-SH); Department of Nuclear Medicine, Koo Foundation Sun Yat-Sen Cancer Center, Taipei, Taiwan (P-IL); Department of Internal Medicine, Koo Foundation Sun Yat-Sen Cancer Center, Taipei, Taiwan (W-TC); and Department of Pediatric Hematology and Oncology, Koo Foundation Sun Yat-Sen Cancer Center, Taipei, Taiwan (R-LC).

## Abstract

Mediastinal nonseminomatous germ cell tumor (MNSGCT)-associated histiocytic proliferations are rare and rapidly fatal disorders. Standard treatment modalities have yet to be established.

We report a case of MNSGCT-associated hemophagocytic syndrome that evolved into malignant histiocytosis/disseminated histiocytic sarcoma (MH/HS), which was initially treated with intravenous immunoglobulin, corticosteroids, and cyclosporine. Then, thalidomide plus cyclophosphamide, adriamycin, oncovin, prednisolone chemotherapy followed by alemtuzumab-containing reduced-intensity allogeneic peripheral blood stem cell transplantation (PBSCT) was used as salvage therapy.

The severe constitutional symptoms and pancytopenia resolved shortly after thalidomide with cyclophosphamide, adriamycin, oncovin, prednisolone. After PBSCT, the patient developed steroid-dependent skin graft-versus-host disease, but maintained a functional life for 1.5 years. Rapid resolution of chronic graft-versus-host disease preceded the fulminant recurrence of hemophagocytic syndrome and MH/HS.

Thalidomide plus chemotherapy followed by alemtuzumab-containing reduced intensity allogeneic PBSCT is effective in allaying MNSGCT-associated histiocytic disorders, but does not prevent eventual relapse. However, further posttransplant immune modulation should be developed to completely eradicate the residual MH/HS cells.

## INTRODUCTION

Histiocytic proliferations associated with mediastinal nonseminomatous germ cell tumor (MNSGCT), ranging from hemophagocytic syndrome (HPS) to malignant histiocytosis (MH)/disseminated histiocytic sarcoma (HS), are very rare. The prognosis of these patients is extremely poor, with survival measured in months, despite various treatments according to Shinoda et al.^1^ Recently, de novo and secondary HS has been successfully treated with thalidomide and alemtuzumab.^2–6^ We report our experience of treating a patient with MNSGCT-associated histiocytic disorders by thalidomide plus cyclophosphamide, adriamycin, oncovin, prednisolone (CHOP) chemotherapy followed by alemtuzumab-containing reduced intensity conditioning before allogeneic peripheral blood stem cell transplantation (PBSCT). This treatment resulted in over 2 years of survival from the onset of histiocytic proliferation, which is the longest reported survival yet.

## CASE REPORT

A 24-year-old man had a history of congenital agenesis of the right kidney and ureteropelvic junction stricture of the left ureter repaired by pyeloroplasty in infancy. He was diagnosed with primary mediastinal mixed germ cell tumor with embryonal carcinoma component without distant metastases in August 2011. Laboratory test results showed elevated α-fetoprotein of 237 ng/mL (normal <20 ng/mL) and normal lactate dehydrogenase of 243 U/L (normal 100–250 U/L). Bleomycin, etoposide, cisplatin (BEP) chemotherapy was given for 4 cycles, followed by removal of the mediastinal tumor plus wedge resection of the right middle and lower lung lobes through mediastinoscopy in January 2012. No residual malignant cells were identified pathologically and therapy was concluded.

**FIGURE 1:**
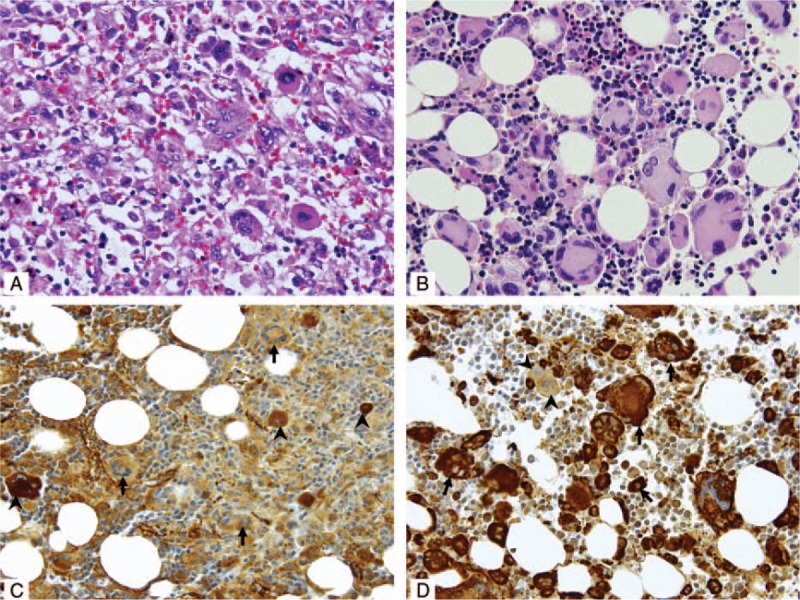
(A) The spleen shows atypical histiocytes with nuclear polylobulation and anaplasia and erythrophagocytosis (H&E stain, 400×). (B) The bone marrow after 4 cycles of thalidomide/CHOP chemotherapy reveals many atypical multinucleated histiocytes (H&E stain, 400×). The atypical histiocytes (arrow) are immunohistochemically (C) negative for factor VIII and (D) positive for CD68. In contrast, megakaryocytes (arrowhead) are positive for factor VIII (C) and negative for CD68 (D).

**FIGURE 2:**
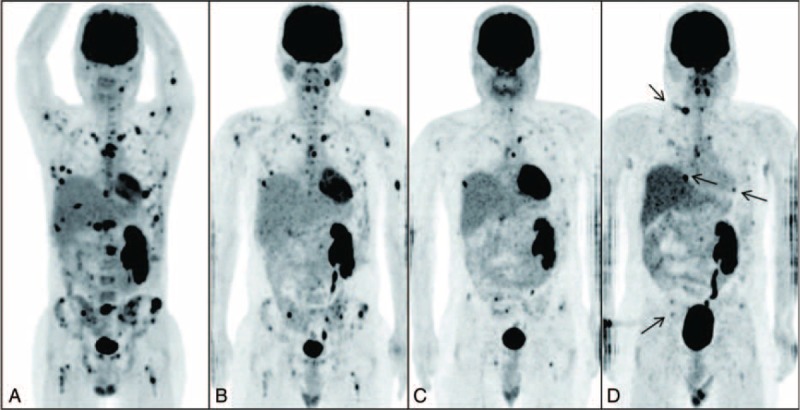
The serial positron emission tomography-computed tomography scans (A) before and (B) after thalidomide/chemotherapy; (C) day +65 and (D) day +300 posttransplant show decreasing [18F]fluorodeoxyglucose (FDG) uptake within lymph nodes in the upper abdominal, paraaortic regions, and also in skull, clivus, left mandible, cervical, thoracic and lumbar spines, pelvic bones, sacrum, sternum, ribs, clavicles, right scapula, bilateral humeri, and bilateral femora. Arrows point at the newly increased FDG-avid lesions in the upper back, right lung, and several bones on day +300 (D) compared with those on day +65 (C) posttransplant.

He developed dry cough in May 2012 followed by progressive constitutional symptoms including generalized pain, fever, night sweats, and weight loss of 4 kg in 1 month. He was admitted in June 2012 for managements of fever of unknown origins, pancytopenia, and splenomegaly. No evidence of germ cell tumor recurrence or any infectious etiology was found. However, multiple skeletal and deep lymph node lesions were found on a positron emission tomography-computed tomography (PET-CT) scan (data not shown). Repeat bone marrow studies showed decreased cellularity and prominent histiocytic hemophagocytosis. Bone marrow cells had a normal 46 XY karyotype, and fluorescence in situ hybridization analyses did not show any abnormalities including isochromosome 12p. He was placed on steroids, intravenous immunoglobulin, and cyclosporine for MNSGCT-associated HPS, beginning in August 2012. His fever was only partially alleviated and he still required high-dose steroids/opioids for symptom control. In addition, pancytopenia persisted, requiring frequent transfusions. He eventually underwent splenectomy on September 28, 2012. The spleen and bone marrow showed diffuse infiltration with bizarre atypical histiocytes and prominent erythrophagocytosis (Figure 1A and B, respectively). By immunohistochemical staining, the atypical histiocytes were negative for CD1a, CD2, CD3, TIA-1, CD20, CD21, CD35, cytokeratin (AE1/AE3), HMB45, and factor VIII (Figure 1C), but positive for CD45, CD68 (Figure 1D), S-100 (focal), lysozyme (focal), CD52, CD30, and CD4 (weak). A PET-CT scan on October 12, 2012 revealed extensive [18F] fluorodeoxyglucose (FDG)-avid lesions (Figure 2A). Thus, MNSGCT-associated MH/HS involving multiple bones, bone marrows, lymph nodes, and spleen was diagnosed. He was transferred to our hospital for further management while still transfusion and high-dose steroid/opioid-dependent.

**FIGURE 3:**
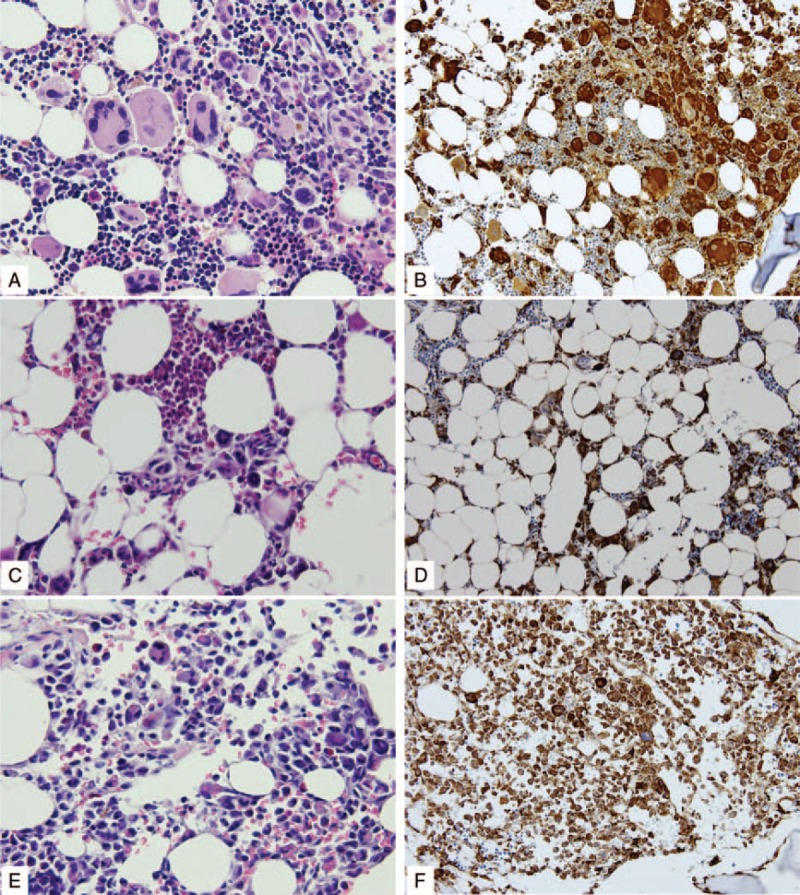
H&E (400×, A, C, and E) and CD68 immunohistochemical (200×, B, D, and F) staining of serial bone marrow biopsies obtained before transplant, but after thalidomide/chemotherapy (A and B), day +301 (C and D), and day +366 after transplant (E and F). As highlighted by CD68 immunohistochemical staining, atypical histiocytes were markedly decreased on day +301 (note the active trilineage hematopoiesis by H&E stain) (D) compared with pretransplant findings (B). In contrast, extensive proliferation of atypical histiocytes with reduced hematopoietic activity was present on day +366 posttransplant (E, H&E stain, 400×; F, CD68, 200×).

Thereafter, the patient was treated with oral thalidomide (50 mg daily) and received 6 cycles of CHOP chemotherapy beginning from October 2012. The treatment resulted in rapid resolution of all constitutional symptoms, establishment of transfusion-independent hematopoiesis, and discontinuation of steroid/opioid. He was able to resume work. However, a follow-up PET-CT scan revealed decreased but still extensive skeletal and lymph node lesions (Figure 2B) on January 14, 2013. Bone marrow studies on March 14, 2013 revealed recovery of normal hematopoietic elements, but presence of multinucleated cells and bizarre atypical histiocytes (Figure 3A), which stained positive for CD52, CD68 (Figure 3B), S-100, and lysozyme. Persistent histiocytic disease was therefore still present.

A decision was made to pursue allogeneic PBSCT as salvage therapy. The conditioning consisted of alemtuzumab (0.2 mg/kg daily from day −14 to day −10), fludarabine (30 mg/m^2^ daily from day −7 to day −3), and melphalan (140 mg/m^2^ on day −2) as modified from the published protocol.^7^ For prophylaxis against graft-versus-host disease (GVHD), the patient received cyclosporine and oral mycophenolate mofetil. On May 8, 2013 (day 0), he received PBSC from an unrelated donor containing 18 × 10^8^ total nucleated cells/kg and 2.3 × 10^6^ CD34^+^ cells/kg. The patient and the donor were HLA-8/8-matched (A 0206/3001, B 1302/5401, Cw 0102/0602, DR 0405/0701) and ABO-mismatched (B into O). Neutrophil engraftment with complete donor chimerism from peripheral blood cells was documented on day +11. He did not require any transfusion after day +11. He was discharged from the hospital on day +19 posttransplant and resumed oral thalidomide (50 mg daily) on day +62 as maintenance. He developed recurrent infections including cytomegalovirus reactivation on day +46 and salmonella O9 (group D1) sepsis on day +82, +144, and +204, respectively, which all resolved under appropriate therapy in an outpatient setting.

However, isolated stage 3 acute skin GVHD developed and prednisolone was added on day +36. Oral mycophenolate mofetil was discontinued on day +41 and cyclosporine was continued through the entire course. Prednisolone was tapered off on day +87 when skin GVHD was stage 1. Prednisolone was restarted 1 week later with gradually increasing dosage as the rash evolved into nonsclerodermatous chronic skin GVHD. He required intermittent high-dose prednisolone (maximum 60 mg/d), but then was placed on daily prednisolone of 25 to 35 mg, with itching rashes waxing and waning. Complete donor chimerism among nucleated peripheral blood cells was documented repeatedly until day +299. Follow-up PET-CT scans on days +65 and +300 revealed marked improvement of FDG-avid lesions compared to pretransplant findings (Figure 2C and D, respectively), although new lesions were noted on day +300. A decrease of abnormal histiocytic proliferation was also documented in bone marrow aspirates on days +61 (data not shown) and +301 (Figure 3C and D). Chimerism studies on bone marrow cells showed mixed chimerism with 4% and 9% recipient cells, respectively.

He developed progressive generalized pain in late April 2014, whereas rapid resolution of skin GVHD after day +355 was noted. He was admitted on account of intolerable pain and air-hunger on May 8, 2014 (day +365). He had spiking fever, hepatomegaly, pancytopenia (neutrophil 0.89 × 10^9^/L, hemoglobin 9.0 mg/dL, platelet count 20 × 10^9^/L), hypertriglyceridemia of triglyceride 272 mg/dL (normal <200), and hyperferritinemia of ferritin 2871 ng/ml (normal 21.81–274.6). Fulminant recurrence of MNSGCT-associated histiocytic proliferation and HPS were documented with prominent bone marrow hemophagocytosis and extensive abnormal CD68+ histiocytic proliferation (Figure 3E and F). Treatment with etoposide/dexamethasone/cyclosporine was administered immediately resulting in transient resolution of fever, pain, and air-hunger. However, the pancytopenia persisted. In addition, the recipient chimerism increased to 15% among peripheral blood cells on day +371 and to 31% among bone marrow cells on day +366. His respiratory function gradually deteriorated. The patient declined endotracheal intubation, which was also in concordance with family wishes. He died on day +401, more than 2 years after the onset of MNSGCT-associated HPS. He had a relatively active life where he continued his career under mostly outpatient care.

## DISCUSSION

Our patient developed HPS within a half year after completion of treatment for MNSGCT, evolving into MH/HS as confirmed by extensive nodal, extranodal (spleen and bone), and systemic (lungs and bone marrow) involvement. MNSGCT-associated hematologic malignancies are thought to result in spread of hematopoietic tumor cells into blood, bone marrow, and extramedullary sites.^8–11^ The clinical course of MNSGCT-associated hematologic malignancies is very aggressive, with 5 (range 0–16) months median survival after diagnosis. Patients die before treatment, do not respond to antileukemic therapy, or achieve only short remissions.^8–11^ In the case of MH/HS, the median survival is even shorter, ranging from a few days to 6 months, with no effective treatment yet available.^1^

Recently, thalidomide has been shown effective in salvage of refractory de novo HS when used alone or combined with chemotherapy.^3–5^ In addition, thalidomide alone has contributed to long-term stabilization in a pediatric case of secondary HS after allogeneic bone marrow transplantation for T-acute lymphoblastic leukemia.^2^ We borrowed the concept and gave the patient thalidomide plus 6 cycles of CHOP which resulted in symptomatic resolution and recovery of hematopoiesis, even though a significant MH/HS tumor load remained, which prompted our decision to consolidate the gains made with chemotherapy by allogeneic PBSC transplantation.

Alemtuzumab—a humanized monoclonal anti-CD52 antibody—has been used as art of reduced intensity conditioning agent for stem cell transplantation in pediatric histiocytic disorders.^7^ Single-agent alemtuzumab has been used in 2 pediatric cases of widespread de novo HS with success.^6^ The malignant cells of MH/HS in those 2 reported patients and in our patient all showed expression of CD52 antigens. Successful allogeneic stem cell transplant for MNSGCT-associated acute megakaryoblastic leukemia and myelodysplastic syndrome, respectively, has been reported.^12,13^ Therefore, we hypothesized that incorporating alemtuzumab in reduced intensity stem cell transplantation might be effective through the direct attack on MH/HS cells, and enhancing the indirect graft-versus-MH/HS immunity.

Our patient had persistent infiltration of MH/HS cells in the bone marrow, but also suffered from prolonged skin GVHD. He achieved disease stabilization for more than a year, which appears to be longer than what has been reported as natural history of the disease. The rapid resolution of skin GVHD activity was concurrent with the fulminant recurrence of HPS as manifested by a massive increase of MH/HS cells. Whether this suggests that graft-versus-MH/HS reaction might play a role in suppressing and containing residual MH/HS cells remains conjectural. We intended to use alemtuzumab followed by donor lymphocyte infusion as salvage therapy for the observed relapse on day +366, but were unable to get alemtuzumab in time from United States. A donor lymphocyte on day +301, when new lesions appeared on PET-CT, might have headed off fulminant disease recurrence, but we did not proceed since we were concerned about GVHD.

The use of thalidomide/CHOP followed by alemtuzumab-containing reduced-intensity PBSCT is the first reported case experiencing much longer survival and good quality of life for a patient with MNSGCT-associated histiocytic proliferative disorders. Further studies are needed to improve the immune modulation strategy toward cure of this devastating condition.
